# Macrophage mechanobiology: from sensing to disease

**DOI:** 10.3389/fimmu.2026.1812501

**Published:** 2026-04-22

**Authors:** Mario D’Ambrosio, Khaled Warasnhe, Mohamed H. Eldesouki, Vikram Joshi, Arthur Beyder, Gianluca Cipriani

**Affiliations:** Enteric Neuroscience Program (ENSP), Division of Gastroenterology and Hepatology, Mayo Clinic, Rochester, MN, United States

**Keywords:** inflammation, macrophage, mechanosensation, mechanotransduction, polarization, stiffness

## Abstract

**Objective:**

Macrophages are highly plastic immune cells that maintain tissue homeostasis through their roles in immune surveillance, repair, and remodeling. Emerging evidence reveals that macrophages, residing in tissues with widely varying stiffness, are profoundly influenced by the mechanical properties of their microenvironment.

**Methods:**

Relevant publications on macrophage mechanoregulation published up to January 2026 were identified through systematic searches of PubMed, EMBASE, and Web of Science using combinations of keywords related to macrophages, mechanosensing, mechanotransduction, and mechanical stimuli (e.g., stiffness, stretch, and shear stress). Articles were screened based on relevance to macrophage biology and mechanistic insights.

**Results:**

Mechanical cues are sensed through mechanosensors such as Piezo1, TRPV4, and integrins, which integrate these signals to regulate immune surveillance, inflammation, tissue repair, and remodeling. Dysregulation of these pathways contributes to the pathogenesis of multiple diseases, including fibrosis, atherosclerosis, and neurodegeneration.

**Conclusion:**

Macrophage mechanobiology represents a critical regulatory axis in tissue homeostasis and disease. Targeting mechanosensing pathways offers promising therapeutic opportunities to modulate inflammation and enhance tissue regeneration.

## 1 Tissue stiffness

Tissues dynamically respond to mechanical cues due to their intrinsic physical properties, such as stiffness, elasticity, viscosity, and plasticity (1–6). Among them, stiffness is the most extensively studied and well-understood physical property that has been investigated for over a century for its association with multiple diseases, including malignancies. Historically, the diagnosis of tumors relied on palpation, a method where physicians would physically assess the consistency and firmness of tissues. This tactile feedback has long suggested a connection between abnormal tissue stiffness and the presence of malignancy or chronic inflammation. The advent of medical technology and development of biomechanics as a field have enabled more precise measurements of tissue stiffness, allowing us to better understand the mechanical properties of diseased tissues. In recent years, tissue stiffness has been recognized not only as a hallmark of disease but also as a mechanistic contributor to disease progression, informing both diagnostic approaches and the development of therapies targeting the altered mechanical microenvironment (7, 8).

**Figure 1:**
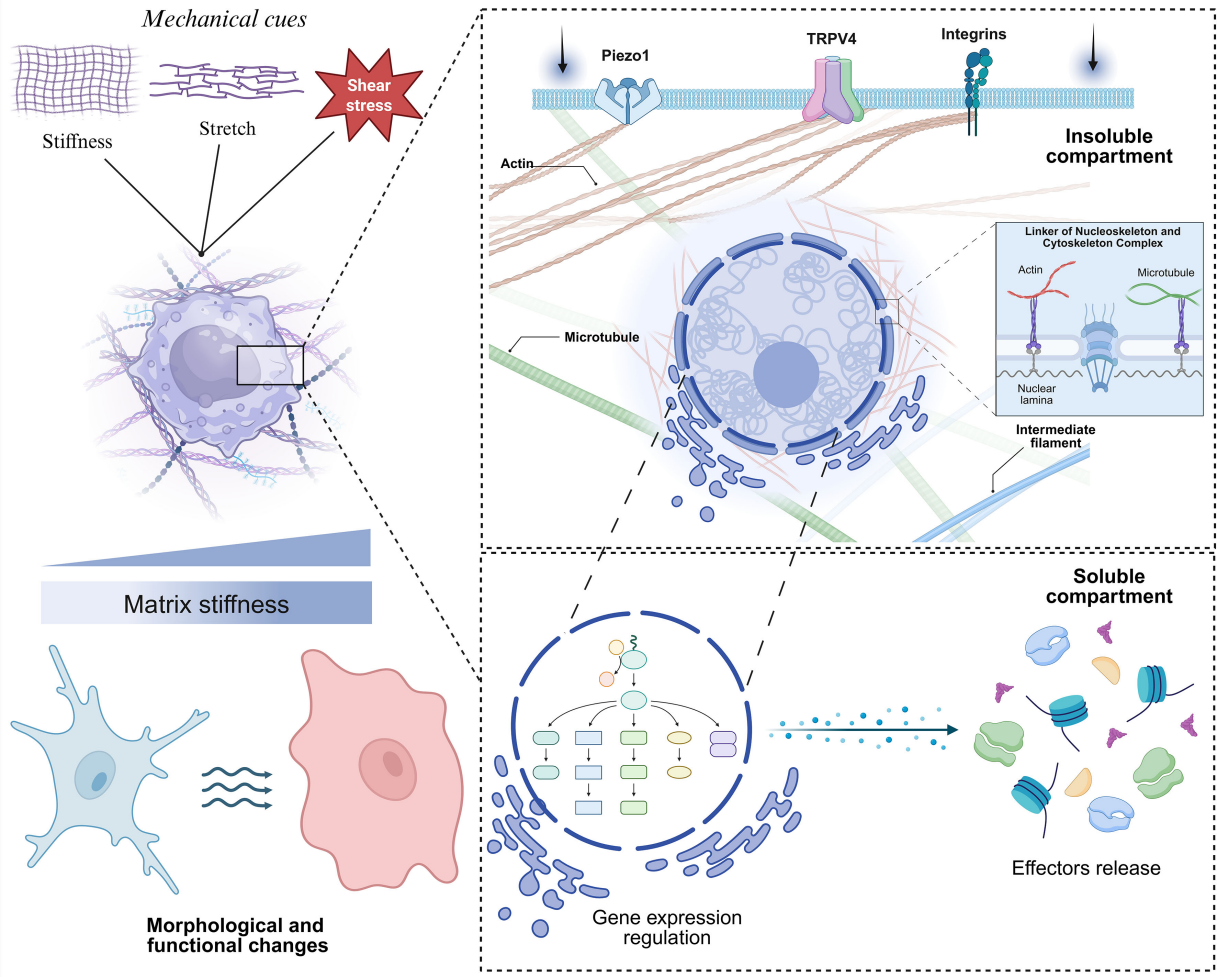
Generic schematic representation of macrophage mechanotransduction. Increased ECM stiffness activates mechanosensitive channels such as Piezo1, TRPV4 as well as integrins. Actin filaments, microtubules, and intermediate filaments interact with the linker of nucleoskeleton and cytoskeleton complex to relay forces to the nucleus, influencing gene expression, macrophage morphology and behavior.

Tissue stiffness refers to the tissue properties that determine how mechanical forces applied to biological tissues (load) lead to shape changes (deformation). In other words, stiffness indicates how much force is needed to cause a specific deformation (9). Tissue stiffness is mostly determined by the extracellular matrix (ECM) and intracellular structures such as the cytoskeleton (**Figure 1**) (1). However, other parts partially contribute to tissue stiffness, such as cell-cell junctions, interstitial fluid pressure, and the organization and density of resident cells (10). ECM is a complex network of proteins and polysaccharides that provide structural and biochemical support to surrounding cells. Its dynamic nature depends on the constant remodeling of its components, ensuring network plasticity and the maintenance of cellular functions within the physiological environment (11). Beyond its structural role, the ECM binds and spatially restricts specific growth factors through proteoglycan interactions and latent complex anchoring (e.g., TGF-β-LTBP complexes), thereby regulating their bioavailability in a protease and force-dependent manner (12). This matrix-mediated control of growth factor presentation contributes to context-dependent regulation of cell differentiation, proliferation, and migration. Myofibroblast contraction activates latent TGF-β1 stored in the extracellular matrix, and this activation requires both mechanical stress and TGF-β1 signaling. Blocking integrins or softening the ECM prevents this activation, suggesting that contraction-dependent TGF-β1 release on stiff matrices promotes localized myofibroblast accumulation and fibrotic progression (11). Latent transforming growth factor−β binding proteins (LTBPs) chaperone secretion of latent transforming growth factor−β (TGF−β) complexes and tether these complexes to the extracellular matrix (ECM) via covalent linkage of LTBP N−termini to matrix microfibrils, thereby targeting activatable TGF−β to specific ECM sites. LTBPs also contain protease−sensitive sites and arginine−glycine−aspartic acid (RGD) motifs that enable regulated release and cell−surface interactions, coupling latent TGF−β deposition to matrix remodeling and localized activation (13). Dysregulation of this balanced system has been implicated in numerous pathological conditions, including fibrosis and cancer (14). In addition to the ECM, the cytoskeleton plays a fundamental role in determining tissue stiffness and cellular mechanical properties. The cytoskeleton is a highly dynamic intracellular network composed of actin filaments, microtubules, and intermediate filaments. Actin filaments, in combination with myosin motors, generate contractile forces to regulate cell shape, cortical tension, and stiffness (15). Microtubules provide structural support and facilitate intracellular transport, while intermediate filaments, such as vimentin or keratins, confer mechanical resilience by bearing tensile stress and maintaining cell integrity under deformation (16, 17). Beyond providing structural support, the cytoskeleton is crucial for mechanotransduction, enabling cells to sense and respond to mechanical cues from their environment by transmitting external forces to the nucleus and activating downstream signaling pathways (18–21). Through these functions, the cytoskeleton not only influences individual cell stiffness but also integrates cellular responses to mechanical forces, shaping cellular functions (22, 23).

## 2 Mechanosensation in macrophages

Among the many cell types capable of responding to mechanical cues, macrophages have emerged as highly mechanosensitive immune cells (24–29). By actively recognizing environmental mechanical cues, such as matrix stiffness, tissue stretch, and compression, macrophages regulate host defense and tissue repair (30, 31). The biological process by which macrophages and other cells detect and respond to mechanical stimuli is called mechanosensation (30, 32). This process is initiated by specialized cellular components known as mechanosensors, which detect and interpret physical cues from the surrounding environment. Among different mechanosensors, the most studied are stretch-activated ion channels (e.g., Piezo1, Transient receptor potential cation channel subfamily V member 4 - TRPV4) and integrins (e.g., αMβ2, α5β1 and αVβ3) (33–38). The spatial distribution of mechano-sensors throughout the cell from the plasma membrane, through the cytoskeleton, to the nucleus and even within organelles like the endoplasmic reticulum enables cells to sense and respond to mechanical stimuli effectively by converting these stimuli into biochemical signals, a process known as mechano-transduction (34–38). This mechano-transductive signaling pathway leads to several changes including gene expression, cytoskeletal restructuring, and ion transport, like calcium, to activate downstream signaling pathways. These processes are critical for normal cellular homeostasis and tissue development, and their dysregulation can contribute to disease pathogenesis (18).

### 2.1 Changes in macrophage morphology and polarization

The morphology of macrophages is highly dynamic and reflects their functional state and ability to interact with other cells during biological processes. In the intestine, for example, mucosal macrophages adopt a more dendritic, elongated morphology suited for sampling luminal antigens and interacting with epithelial and immune cells, whereas muscularis macrophages display a stellated or spindle-like shape aligned with smooth muscle fibers, supporting neuromuscular function and tissue integrity (39, 40). Elongation is typically associated with an increased migratory capacity, facilitating macrophages engraftment into target tissues, whereas an increase in cell surface area facilitates their phagocytic activity by enhancing target engagement and engulfment (25, 41).

**Figure 2:**
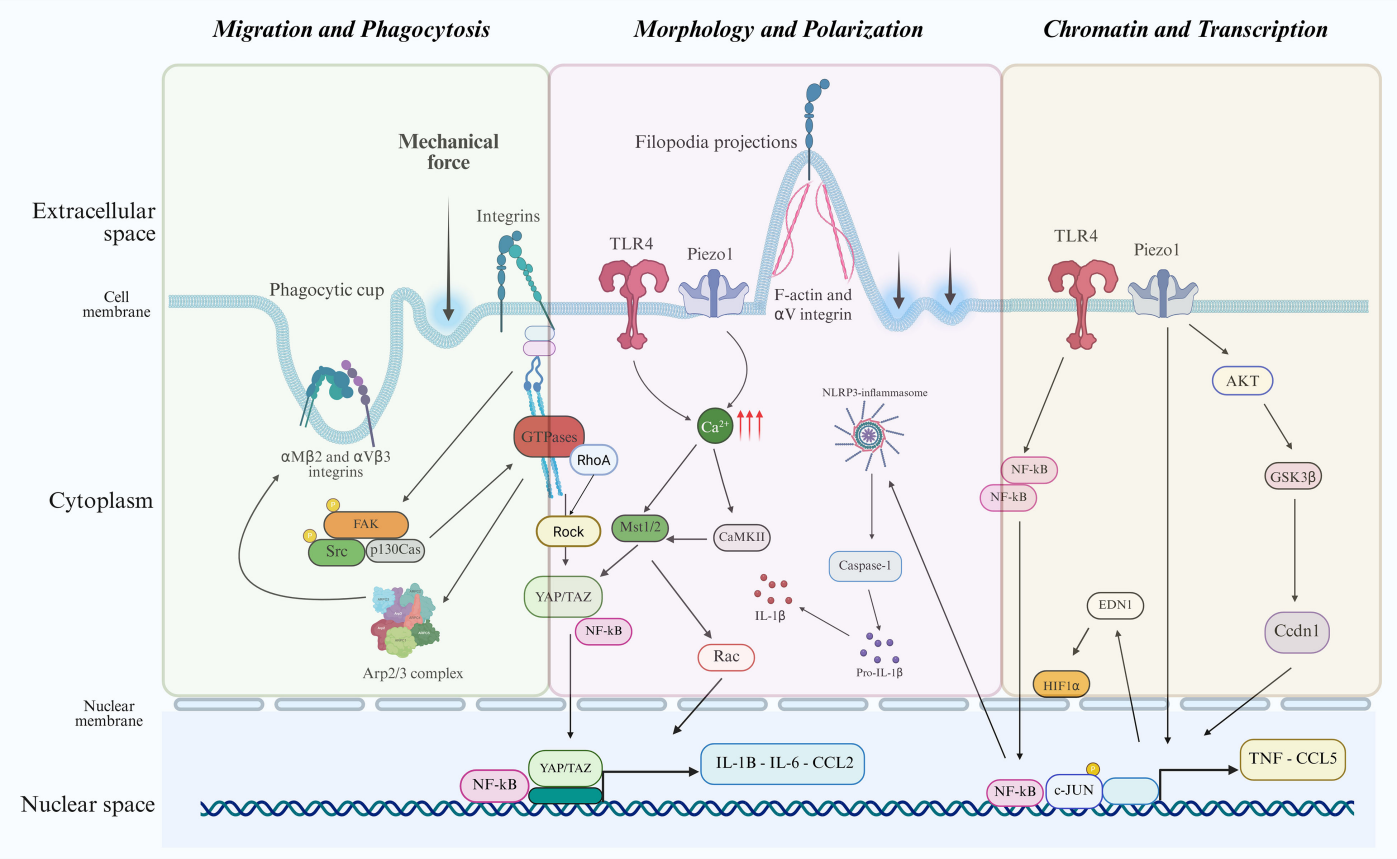
Schematic of how mechanical force acting on the cell membrane engages multiple mechanosensitive channels (e.g. Piezo1 and integrins). Mechanical-dependent Ca^2+^ influx activates downstream signals including CaMKII and Mst1/2; NF-κB and JAK–STAT; NLRP3 inflammasome activation and Caspase-1 activation; actin remodeling (Tmod1, Arp2/3) and adhesion signaling (FAK–Src–p130Cas, small GTPases). Crosstalk with AKT–GSK3β, Rac, HIF-1α, c-JUN, YAP/TAZ and Cyclin D1 further shapes transcription. Net outputs include pro-inflammatory gene induction and programs supporting proliferation, survival, and metabolic reprogramming.

The morphology of *in vitro*-polarized macrophages is a characteristic hallmark of their polarization state. Pro-inflammatory (LPS/IFN-γ-treated) macrophages tend to be more rounded and flattened, while anti-inflammatory (IL-4/IL-13-treated) macrophages assume an elongated shape (25). A combination of *in vitro* and *in vivo* studies demonstrates that different degrees of stiffness influences macrophage morphology and polarization (**Figure 2**) (42). Substrate stiffening enhances integrin clustering and focal-adhesion maturation, driving recruitment and activation of FAK and Src, which stimulate RhoA/ROCK-mediated actomyosin contractility. Increased actin stress fibers and myosin-II tension raise intracellular and perinuclear forces, promote nuclear envelope deformation, and enable nuclear translocation/activation of mechanosensitive transcriptional regulators (e.g., YAP/TAZ and NF−κB). These biophysical changes both change cell morphology (spread, polarized shapes with prominent stress fibers) and reinforce transcriptional polarization programs: altered nuclear mechanics and chromatin accessibility stabilize proinflammatory gene expression downstream of TLR4 and other receptors (20, 25, 43).

Among various sensors capable of converting mechanical stimuli into biochemical signals that regulate morphology, the cytoskeleton emerged as a central player (44, 45). On softer substrates, macrophages typically exhibit a rounded shape with localized F-actin and αV integrin staining; in contrast, stiffer substrates induce a more spread morphology with filopodia projections, pronounced F-actin organization, and enhanced αV integrin staining (25). Inhibition of cytoskeletal dynamics (disrupting actin polymerization or myosin-II contractility) prevents focal-adhesion maturation and integrin/FAK–Src–RhoA/ROCK–driven tension, reducing mechanosensitive NF-κB activation and TLR4-dependent signaling (p-IκBα, p-NF−κB p65 translocation) and thereby lowering proinflammatory cytokine production and impairing macrophage polarization on stiff substrates (26). These biomechanical responses suggest that macrophage shape is a readout of the mechanical environment reflecting their functional state.

Macrophage phenotypes are shaped by a variety of factors, including cellular origin and environmental cues (46–48). Although the M1/M2 paradigm, pro- and anti-inflammatory states, has traditionally been used to classify macrophages, recent studies have revealed a much broader phenotypic spectrum (49, 50).

While the dynamic response of macrophages to biochemical signals is well established, their sensitivity to biophysical cues has only recently gained attention. Generally, stiffer substrates and mechanical stretching, which may reflect general properties of damaged/inflamed tissues, promote M1-like polarization, characterized by increased inflammatory cytokine production and effector function. In contrast, softer substrates and cyclic strain, which may reflect properties of healthy/normal tissues, favor M2-like polarization, supporting tissue repair and inflammation resolution (28). Although these effects are mediated through various mechanosensitive pathways, Piezo1 channel has been primarily studied for its significant role in macrophage mechanotransduction (**Figure 1**) (51, 52). Piezo1 activation with the selective agonist Yoda1 induces Ca^2+^ influx and drives M1 polarization, characterized by increased expression of pro-inflammatory markers and cytokine production (30, 53). *In vivo* application of PEG-based hydrogels, mimicking increased stiffness, activated pro-inflammatory macrophage responses in a Piezo1-dependent manner, while softer substrates led to reduced activation and a milder foreign body response. Similarly, macrophages cultured on soft matrices produced less pro-inflammatory mediators than those on stiff substrates upon lipopolysaccharide (LPS) stimulation (26). Toll Like Receptor 4 (TLR4), a pattern recognition receptor, has been implicated in this mechanotransduction by forming a complex with Piezo1, which promotes calcium influx and activates the CaMKII-Mst1/2-Rac signaling axis (26).

Cyclic stretch induces IL-1β release in mouse alveolar macrophages via NLRP3 inflammasome activation involving caspase-1 and TLR4 pathways (54). It also activates focal adhesion kinase (FAK) and NF-κB during stretch-induced M1 polarization (55). Human monocyte-derived macrophages exposed to shear stress similarly increase secretion of Tumor Necrosis Factor-alpha (TNF-α), IL-8, and other pro-inflammatory mediators (**Figure 2**) (56).

**Figure 3:**
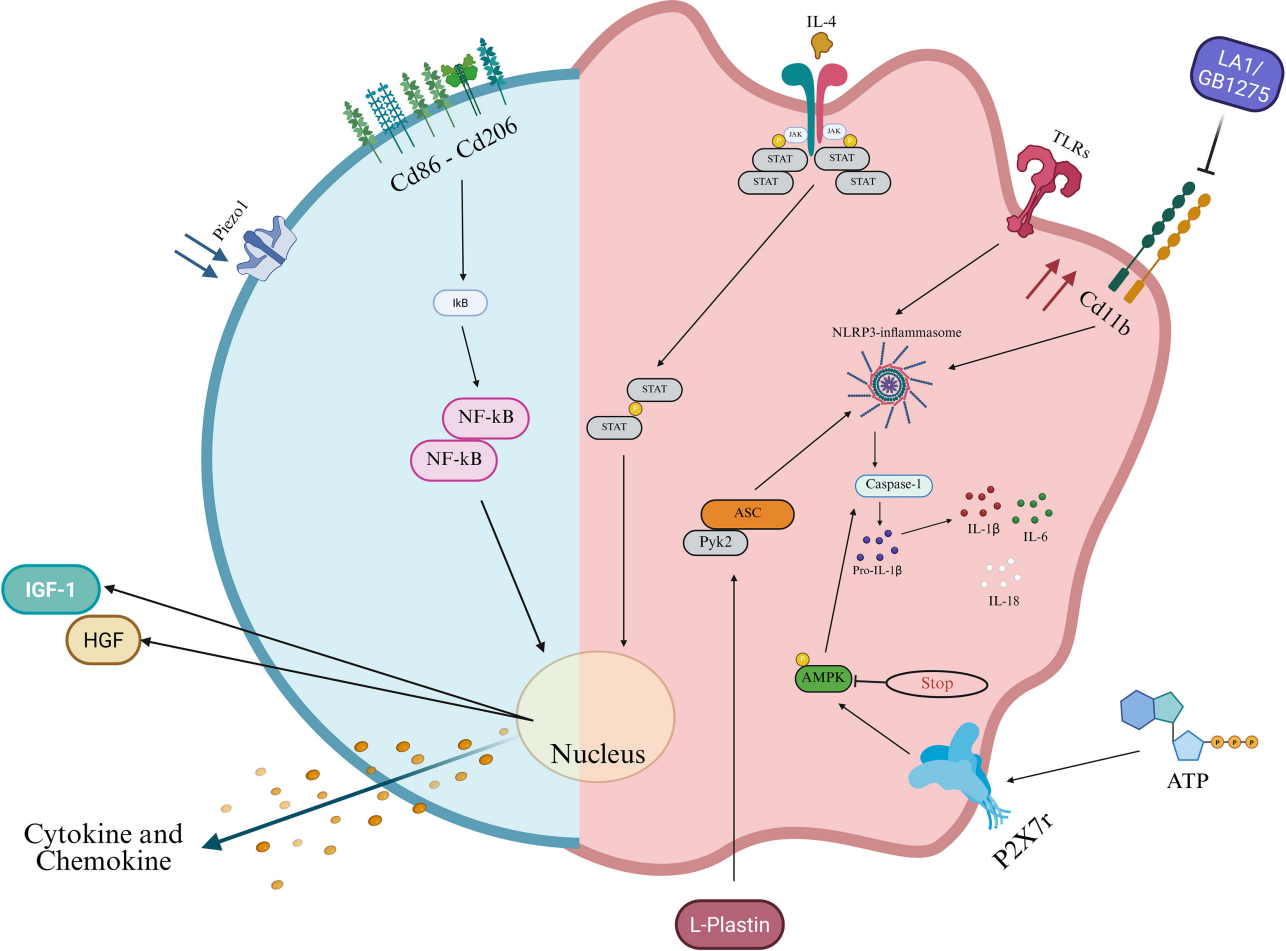
Mechano-dependent pathways driving anti−inflammatory activation of macrophages. Schematic showing how mechanical cues sensed at the membrane (Piezo1, integrin/focal adhesion complexes) and transmitted through the cytoskeleton (L−plastin, Rho GTPases, Pyk2) and calcium/kinase signaling converge on nuclear transcriptional programs that promote anti−inflammatory, pro−resolving phenotypes (upregulation of CD206, secretion of IGF−1, HGF and other pro−resolving cytokines/chemokines). Parallel pro−inflammatory inputs (TLRs, ATP → P2X7R → Ca2+ influx) activate the NLRP3 inflammasome (ASC → caspase−1 → IL−1β, IL−18) and induce IL−6; AMPK provides inhibitory control of inflammasome activation. Cytokine receptor signaling (IL−4 → JAK/STAT) cooperates with mechanotransduction to bias transcription toward resolution. Solid arrows indicate activation/flux; dashed arrows indicate inhibition or negative regulation.

However, it is worth noting that many studies also have reported effects opposite to those described above, with induction of a more protective, anti-inflammatory phenotype (**Figure 3**). For example, in murine bone marrow-derived macrophages (BMDMs), low substrate stiffness promotes M1 polarization, whereas higher stiffness induces M2 characteristics via a reactive oxygen species (ROS)-mediated NF-κB signaling pathway (57). In another study, compliant hydrogels (polyacrylamide hydrogels of varying elastic moduli) enhanced NLRP3 activation, pyroptosis, and IL-1β/IL-6 release after LPS priming and nigericin (antibiotic, well-known activator of the NLRP3 inflammasome) treatment, compared to stiffer matrices (58). Mechanical stimulation also promoted M2 polarization via IL-4/JAK/STAT signaling in a tendon-bone healing model (59) and macrophages showed different responses to uniaxial versus biaxial strain (60, 61). However, it is important to remember that macrophages isolated from tissues may respond to mechanical stimuli differently than BMDM due to their distinct developmental origins and local mechanical conditioning (62).

Increased matrix stiffness is also reported to suppress macrophage-mediated inflammation. Using a bleomycin-induced lung fibrosis model, the authors showed that L-plastin is required for macrophage activation and inflammatory responses. They identified a novel mechanism in which L-plastin stabilizes Pyk2-ASC interactions to promote NLRP3 inflammasome assembly; this mechanism is induced by mechanotransduction (63). Recent work by Joshi et al. directly compares substrate stiffness to pharmacologic activation of CD11b cells and shows that macrophages on very soft collagen gels (0.2 kPa) exhibit enhanced NLRP3-dependent IL-1β production, whereas treatment with the CD11b agonist leukadherin-1 (LA1/GB1275) recapitulate a stiff-substrate effect by suppressing NLRP3 assembly and IL-1β release. Notably, LA1 also reduced IL-6 and altered several surface-marker programs, but did not recapitulate all stiffness-dependent changes in differentiation or marker expression, indicating that integrin agonism can overlap with but is not identical to bulk matrix stiffening (64). Cyclic stretch was found to inhibit ATP-stimulated IL-1β release in LPS-primed macrophages by suppressing AMP-activated protein kinase (AMPK) phosphorylation and caspase-1 activity, independent of NF-κB, suggesting a role for stretch in preventing excessive inflammasome activation and maintaining immune homeostasis (65, 66). Another study demonstrated that both static and cyclic stretch suppressed LPS/IFNγ-induced inflammation in BMDMs, while IL-4/IL-13 responses were suppressed by cyclic but enhanced by static stretch. Both types of stretch increased CD11b expression and decreased Piezo1 levels; siRNA knockdown of either molecule abrogated these effects (67). Additionally, when macrophages are exposed to a mechanical stretch in the skin, they adopt an M2 phenotype and release growth factors such as hepatocyte growth factor (HGF) and insulin-like growth factor 1 (IGF-1), promoting stem cell activation and hair regeneration (68).

Beyond sensing niche stiffness and cyclical or acute stimuli, macrophages also respond to physical cues such as biomaterial surface topography like grooves, micropatterns, or wrinkled surfaces in a range of different ways, sometimes discordant. They can elongate and polarize toward an M2 phenotype, which enhances pro-healing markers while reducing pro-inflammatory cytokine secretion (26, 69). Kosoff et al. (70) showed that microtopographic patterns alter macrophage morphology and inflammatory outputs, with responses shaped by surface presentation and adsorbed proteins. Malheiro et al. (71) demonstrated that nanotopography alone does not dictate phenotype—surface chemistry and protein conditioning determine whether topography promotes pro-inflammatory or pro-healing programs. Monteiro et al. (72) similarly show evidence that topography interacts with material chemistry and soluble cues to produce discordant macrophage outcomes, underscoring the need to consider multiple contextual factors. These observations broaden the view of macrophages mechanosensitivity, underlying that different physical cues can shape their functional state. In summary, mechanical cues drive macrophage phenotypic changes across a broad spectrum, influenced by numerous factors summarized in **Table 1**.

**Table 1:** Summary table of selected studies included in this review, outlining experimental systems, mechanical cues, signaling pathways, and associated cellular and functional outcomes.

Cell source/mouse model	Mechanical microenvironment	Mechanical Input	Macrophages’ response	References
RAW 264.7, BMDMs, THP-1, primary macrophages: fibrosis model	PEG-RGD hydrogels; polyacrylamide gels (2D compliant substrates, ECM-coated)	0.2–323 kPa (elastic modulus)	Context-dependent stiffness response: soft matrices generally reduce inflammation and promote M2 phenotypes (biomaterials, repair) but can also enhance NLRP3 inflammasome activation (IL-1β); conversely, increased stiffness or integrin activation (e.g., CD11b agonism) can suppress inflammasome assembly and inflammatory cytokine release. Mechanistically, stiffness-induced signaling involves cytoskeletal regulators (e.g., L-plastin) and integrin-dependent pathways that modulate Pyk2–ASC interactions and NLRP3 activation	(24, 27, 57, 58, 73, 74, 77, 78, 100)
BMDMs, THP-1, U937	Micropatterned substrates (line confinement) and ECM-coated elastomeric membranes (collagen I, RGD peptides)	20-50 μm confinement; uniaxial vs biaxial strain (Flexcell systems)	Cell shape/ECM cues bias toward M2 or immune tuning: elongation promotes M2 polarization, while ECM composition and geometry modulate epigenetic and inflammatory programs	(25, 43, 60, 69)
BMDMs and knockout mice	Polyacrylamide hydrogels ± Piezo1 agonist (Yoda1); stiffness-controlled culture	5–40 kPa; Yoda1 (5 μM)	Piezo1 integrates mechanical signals into inflammatory or regenerative outputs that promotes pro-inflammatory Ca^2+^ signaling (TLR4-CaMKII-Rac1), but can suppress angiogenesis and regulate proliferation depending on context	(26, 29, 30, 96)
Alveolar macrophages, RAW264.7, BMDMs	Flexible silicone membranes (BioFlex/Flexcell stretch systems, ECM-coated)	Cyclic stretch 5-20% strain, 0.5 Hz (minutes-hours)	Stretch produces divergent outcomes: can induce M1/NLRP3 activation (injury models) or suppress inflammasome/IL-1β and modulate healing phenotypes depending on frequency, duration, and context	(54, 55, 65, 67)
Monocytes, BMDMs	Agarose-embedded cells in multi-axial bioreactor (shear + compression loading)	Shear ±25° rotation at 1 Hz; compression ~10-20% strain (1h-3 days)	Shear/compression consistently promote pro-inflammatory activation (IL-6, TNFα, MIP-1α), particularly in remodeling or injury contexts	(56)
Tissue (skin, rotator cuff [RC] repair)	*In vivo* mechanical stimulation (skin-stretch device; treadmill loading)	Stretch up to ~40% (days); treadmill 5–10 m/min (repeated cycles)	Physiological mechanical loading promotes M2-like repair programs, driving IL-4/JAK-STAT signaling and growth factor-mediated regeneration (HGF, IGF-1)	(59, 68)
BMDMs, THP-1, primary macrophages	Phagocytic platforms (polyacrylamide beads, DAAMPs microparticles, ligand-coated hydrogels)	Target rigidity (soft vs stiff particles, kPa range); dynamic traction forces	Target mechanics regulate phagocytosis: stiff targets enhance integrin-dependent engulfment (Rac1, talin/vinculin), while soft targets slow or alter uptake, highlighting a mechanosensitive checkpoint	(79, 80, 85–87)
BMDMs and tissue resident macrophage	Oscillatory shear stress on fibronectin-coated substrates (flow/pressure system)	40–60 mmHg oscillatory pressure	PIEZO1-mediated mechanosensing drives strong pro-inflammatory programs (c-JUN-EDN1-HIF1α), linking mechanical stress to host defense and pathology	(95)

### 2.2 Effect of forces on macrophage migration and phagocytosis

Macrophages are highly plastic cells whose ability to migrate, and phagocytose is essential for immune surveillance, tissue remodeling and host defense (73, 74). Mechanical forces influence migration by activating mechanosensitive signaling pathways that regulate cytoskeletal organization, cell adhesion, and surface receptor expression (30, 75). For instance, fluid shear stress enhances macrophage motility through cytoskeletal rearrangement and focal adhesion modulation (76). Pathway analysis of macrophages cultured on stiff substrates revealed the upregulation of genes associated with cell migration and an increase in the distance traveled by migrating cells. Mechanistically, macrophages sense increased tissue stiffness and respond by enhancing cytoskeletal rearrangement through Tropomodulin 1 (Tmod1) (77). Notably, genetic deletion or inhibition of Tmod1 in macrophages reduced their motility, leading to a decrease in atherosclerotic plaque formation, thereby suggesting a mechanistic link between ECM stiffness, macrophage migration, and disease progression (77). However, opposite results have been reported using a human macrophage cell line (THP-1), where migration was reduced on stiffer substrates compared with softer ones, highlighting potential differences between primary and immortalized macrophages or species-specific responses (78).

Studies using polyacrylamide gels of varying elastic moduli have demonstrated that macrophage phagocytic capacity increases on stiffer substrates (27). For example, when BMDM or human monocyte-derived macrophages (hMDMs) are cultured on stiffer matrices (e.g., >20 kPa), they show enhanced uptake of opsonized particles compared to those on softer substrates (e.g. ~1 kPa) (79, 80). This increased phagocytic activity is attributed to several interconnected mechanisms. Stiffer substrates promote clustering of integrins, such as αMβ2 (Mac-1) and αVβ3, leading to the formation of robust focal adhesions (80). These adhesions anchor the actin cytoskeleton to the ECM, providing stable traction points necessary for force generation during phagocytosis. Substrate stiffness leads to the activation of FAK and Src kinases family (SFKs), which phosphorylate downstream targets involved in actin remodeling, such as paxillin and p130Cas. This signaling cascade activates Rho family GTPases (RhoA, Rac1, Cdc42), promoting actin nucleation and branching via the actin-related protein 2/3 (Arp2/3) complex, essential for phagocytic cup formation and closure (**Figure 2**) (81–83). On stiff substrates, macrophages exhibit greater myosin II-mediated contractility, generating cortical tension that stabilizes the phagocytic cup and facilitates target internalization. Inhibition of myosin II (e.g., with blebbistatin) or culture on compliant substrates reduces phagocytosis efficiency, underscoring the importance of actomyosin-generated forces (84, 85). Macrophages form an adhesion-like mechanosensitive complex during phagocytosis, involving talin and vinculin, which link integrins to the actin cytoskeleton. Substrate stiffness enhances the recruitment and activation of these proteins, stabilizing the interface between the macrophage membrane and the target to withstand engulfment forces (80, 86, 87).

### 2.3 Change in chromatin and transcription regulation

Mechanical forces and stiffness of the cellular environment are increasingly recognized as key regulators of epigenetic state (88). These biomechanical cues can influence gene expression and cellular behavior by modulating the chemical modifications to DNA and histone proteins, such as methylation, acetylation, and chromatin remodeling, without altering the underlying genetic code (89–91). Cells experiencing changes in matrix stiffness or mechanical strain undergo shifts in nuclear architecture, chromatin accessibility, and transcriptional activity. This mechanosensitive epigenetic reprogramming plays a fundamental role in processes such as stem cell differentiation, immune cell activation, and tissue adaptation, and has been implicated in the progression of fibrosis, cancer, and aging-related diseases (92, 93). Understanding how physical forces shape the epigenetic landscape provides critical insight into how cells integrate mechanical and molecular signals to guide functional outcomes.

Mechanical forces reshape the cytoskeleton of macrophages, which in turn affects nuclear morphology, positioning, and the localization or activity of transcriptional regulators, ultimately altering gene expression (94). Piezo1 serves as a key mechanosensor that responds to cyclical hydrostatic pressure by activating c-JUN and upregulating Endothelin-1 (EDN1), which stabilizes the Hypoxia Inducible Factor 1-alpha (HIF1α) and triggers a sustained proinflammatory transcriptional program (95). Similarly, Piezo1 activation, via increased stiffness or the chemical agonist Yoda1, leads to reduced fibroblast growth factor 2 (FGF2) production in BMDMs and amplifies inflammatory responses (96). Mechanical cues also directly regulate transcription factors like YAP/TAZ (Yes-associated protein and transcriptional coactivator with PDZ-binding motif), which are activated by substrate stiffness and shear stress (97). YAP/TAZ activation enhances the expression of genes involved in macrophage activation, polarization, and immune responses (**Figure 2**) (32, 98). YAP depletion suppresses macrophage inflammation, whereas overexpression of active YAP increases it. *In vivo*, soft substrates reduce inflammatory marker expression and YAP activation compared to stiff materials (97).

Beyond transcription factor regulation, mechanical stimuli influence chromatin structure and epigenetic states. Substrate stiffness modulates chromatin remodeling and histone modifications, altering DNA accessibility, and transcriptional activity in macrophages (90).

Deletion of YAP/TAZ suppresses pro-inflammatory gene expression and promotes reparative pathways, as evidenced by downregulation of IL-6 and upregulation of arginase1 (Arg1) through disruption of the HDAC3-NCoR1 repressor complex (32).

Consequently, YAP/TAZ deletion attenuates fibrosis and hypertrophy, improves angiogenesis, and enhances cardiac function after myocardial infarction, whereas YAP activation exacerbates these outcomes (97).

Mechanical forces also interact with soluble cues, such as cytokines and growth factors, to modulate transcription synergistically or antagonistically. For instance, Piezo1 upregulation in periodontal tissues and BMDMs drives macrophage proliferation via the AKT/GSK3β-Ccnd1 signaling axis (29, 99) (94). In a recent study, Meizlish et al. (2024) showed that inhibiting cytoskeletal rearrangement with latrunculin opens chromatin regions for transcription factors promoting Fizz1 (also known as RetnlB), RetnlA, and Arg1 expression in BMDMs (29).

## 3 Clinical implications of mechanical force on macrophages

**Figure 4:**
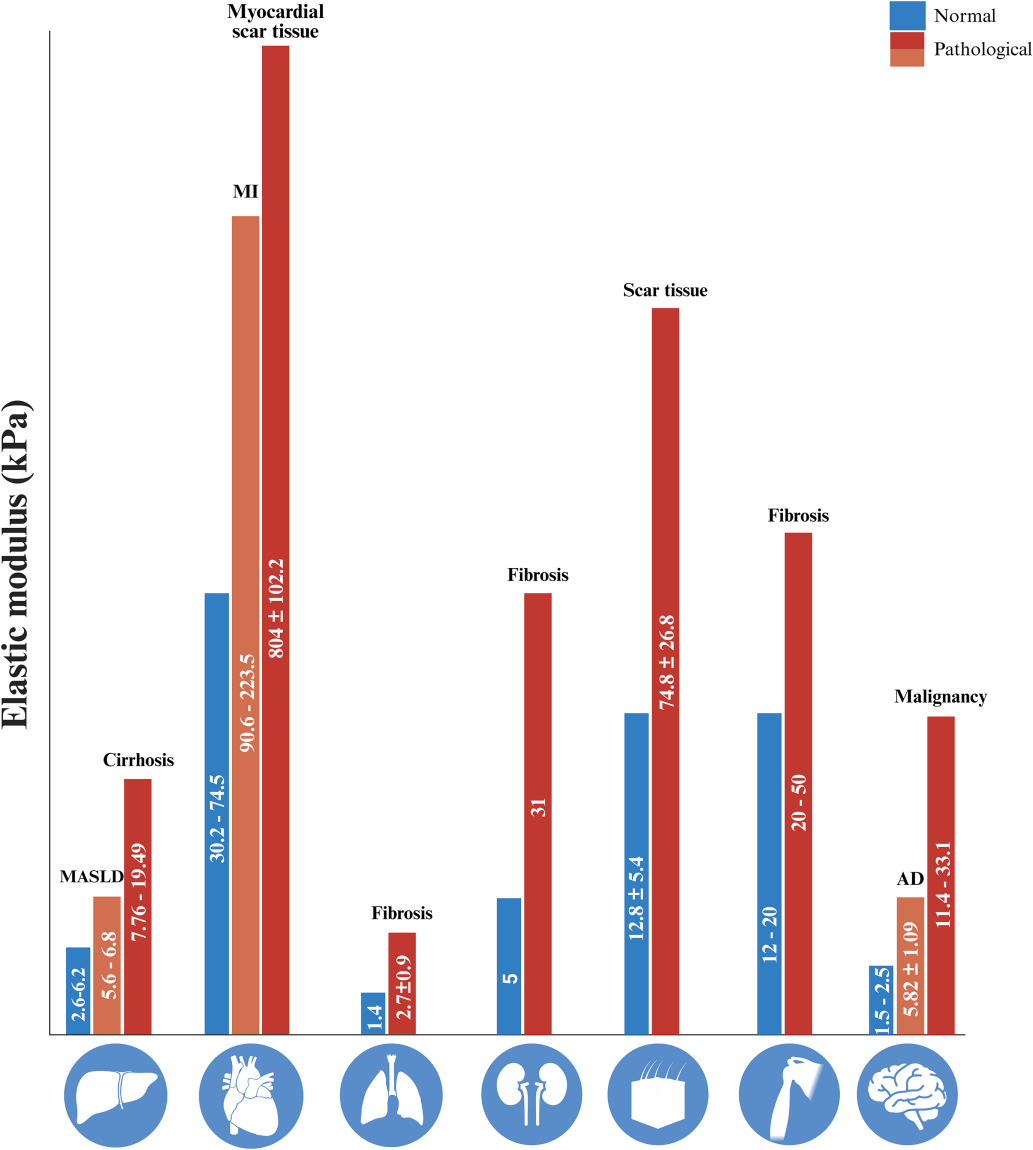
Elastic modulus (kPa) of normal and pathological tissues across different organs. Blue bars represent normal tissue stiffness, while red/orange bars represent pathological tissue stiffness.

In the beginning of the review, we pointed out the “tumor-” stiffening of cancerous tissues. In fact, mechanical properties are common hallmarks of many biological processes in both health and disease. As organs become diseased or aged, their tissue stiffness and other mechanical properties change significantly (**Figure 4**). These alterations occur alongside a range of biological processes, such as inflammation, metabolic reprogramming, immune cell infiltration, and ECM remodeling, which together shape the local microenvironment (100, 101). Macrophages, as key sensors and responders to both mechanical and biochemical cues, adapt their activation, polarization, and function accordingly as described in the previous sections. Separating the specific contribution of mechanical forces from other concurrent signals remains challenging, but it is increasingly clear that mechanotransduction plays a critical role in regulating macrophage behavior across a range of pathological contexts, including fibrosis, cancer and atherosclerosis (81, 100).

While mechanical stress is a shared feature of diseased and aging tissues, the specific mechanotransduction pathways activated in macrophages can vary significantly across different organ systems. In the heart, cardiomyopathies, which account for approximately 5% of all myocardial disorders, lead to stiffening of cardiac tissue due to ECM accumulation and amyloid proteins deposition, disrupting the balance of resident macrophages and promoting recruitment of inflammatory CCR2^+^ monocytes (102–107). Outside the heart, in the vascular system, hypertension and related conditions modulate this shift and contribute to pathological remodeling (108). In atherosclerosis, macrophages are exposed to hemodynamic forces such as shear stress, sensed by Piezo1, which enhances monocyte transmigration, cytokine production (e.g., IL-1β, IL-6), and matrix metalloproteinase activation, destabilizing plaques (109). Notably, macrophage accumulation in plaques relies more on local proliferation of resident macrophages than monocyte infiltration (110). During macrophages foam cell transition, stiffness sensing promotes foam cell proliferation and uptake of oxidized LDL (oxLDL) (111). Following ischemia, increased tissue stiffness modulates remodeling and angiogenesis through Piezo1–CaMKII–ETS1 mediated signaling (96, 112–116). In heart failure, macrophages may influence myocardial remodeling in response to mechanical load via TRPV4 activation. While beneficial in some contexts, TRPV4 activation, based on parallels with neuronal injury, may also drive inflammation by promoting the release of different interleukins (e.g., IL-1α and IL-1β) (117).

In the brain, emerging evidence strongly associates microglia, the resident immune cells of the brain, in the progression of Alzheimer’s disease (AD), which is primarily characterized by the accumulation of amyloid-beta (Aβ) plaques. One of the defining features of this disease is the localized increase in tissue stiffness, resulting from protein aggregation and ECM remodeling. These changes, which plausibly alter tissue stiffness characteristics (intra-tissue heterogeneity between PAT and NPAT compartments), can significantly alter the mechanical landscape encountered by microglia, triggering mechanotransduction pathways that influence cellular responses (118–121). The key mechanosensor expressed by microglia is Piezo1, which is sensitive to membrane tension and substrate rigidity. In regions enriched with Aβ42, the more aggregation-prone form of amyloid, Piezo1 is upregulated and facilitates Ca^2+^ influx, acting as a critical mediator of downstream signaling pathways. Calcium signaling, in turn, regulates essential microglial functions, including cytokine release (e.g., TNF-α and IL-6), actin cytoskeleton remodeling, migration, and phagocytic activity, all of which are crucial for the containment and clearance of pathological aggregates (121, 122). Importantly, pharmacological activation of Piezo1 using the agonist Yoda1, mimic these effects, promoting Ca^2+^ entry into microglia (123). Conversely, conditional deletion of Piezo1 specifically in microglia has been shown to exacerbate Aβ plaque accumulation, impair microglial function, and cause cognitive deficits in mouse models (123). Beyond Piezo1, other mechanosensitive components, including TRPV4, integrins, and cytoskeletal-associated molecules, are increasingly recognized as part of the mechanosensory toolkit that governs microglial responses to altered brain biomechanics (124). Integrins serve as key links between the ECM and the cytoskeleton, mediating adhesion, migration, and outside-in signaling in response to matrix stiffness and tension (125, 126). The integration of signals from these mechanosensors likely contributes to the functional polarization of microglia, influencing whether they adopt pro-inflammatory (neurotoxic) or anti-inflammatory (neuroprotective) roles.

In the lungs, alveolar macrophages respond to cyclic stretch and elevated stiffness through TRPV4. TRPV4 activation leads to increased calcium influx, which promotes macrophage motility and phagocytosis. It also increases the production of ROS and reactive nitrogen species (RNS), resulting in increased endothelial permeability (127–129). Growing evidence suggests that TRPV4 plays also a role in the initiation and development of acute lung injury/acute respiratory distress syndrome (ALI/ARDS) by promoting endothelial calcium influx and barrier disruption (130, 131). In addition, high tidal volume during mechanical ventilation activates TLR4, leading to increased calcium influx, which drives oxidative stress and inflammasome activation (ASC-NLRP3-Caspase-1) with subsequent IL-1β release, all key in ventilator-induced lung injury and fibrosis (54, 132, 133). Pulmonary fibrosis occurs due to cyclic epithelial injury and repair, leading to excessive inflammation and fibroblast secretion of the ECM which are pro-fibrogenic and pro-inflammatory (134–136).

The liver’s unique microvascular structure and blood flow dynamics add another layer of mechanical regulation (137). The shear stress can significantly influence macrophage behavior (138). Liver sinusoids in Metabolic dysfunction–Associated Steatotic Liver Disease (MASLD) undergo structural alterations mediated by mechanotransduction pathways (139). In response to these stimulus LSECs lose their capability to control bioavailability of NO leading to HSCs activation that releases chemokines such as macrophage colony-stimulating factor (CSF1) and monocyte chemoattractant protein-1 (MCP1), which promote macrophage adhesion, migration, and cytokine secretion altering the balance of liver inflammation and injury (140, 141).

Despite these exciting findings, a comprehensive understanding of how mechanical forces regulate macrophage behavior across diverse tissues is still developing, and many organs have been only marginally studied in this context. For instance, Shen and colleagues recently reviewed the role of macrophages in hypertrophic scarring (HS), a fibrotic condition where increased ECM stiffness alters macrophage polarization, impacting both inflammation and tissue repair; however, the exact mechanisms by which these macrophages changes contribute to fibrosis remain incompletely elucidated (142). Recent studies suggest that kidney macrophages can sense matrix stiffness and mechanical stress, thereby contributing to fibrosis and the progression of chronic kidney disease (143). Adipose tissue provides another example where macrophages may integrate mechanical and metabolic cues. During obesity, persistent overnutrition induces white adipose tissue (WAT) hypertrophy, where mechanical stress and hypoxia trigger not only adipocyte death but also activation of adipose tissue macrophages (ATMs) (144, 145); while this premise is increasingly recognized, the field is still working to provide clear mechanistic evidence for this integration.

Targeting mechano-driven pathways responsible for macrophage changes has emerged as a promising therapeutic strategy in these contexts (146). In the cardiac tissue, where Piezo1-mediated signaling regulates macrophage proliferation and contributes to maladaptive remodeling, Piezo1 antagonists (e.g., GsMTx4, Dooku1 and salvianolic acid B) reduce cardiac fibrosis in murine models and may potentially be used as antifibrotic agents (147, 148). Similarly, mechanical ventilation can drive alveolar macrophage–mediated inflammation in the lungs through TRPV4 activation, and pharmacological inhibition of this channel has shown promise in preclinical models of acute lung injury (149, 150). Beyond ion channels, integrin-mediated focal adhesion signaling through FAK and downstream YAP/TAZ activation enables macrophages to sense and respond to ECM stiffness, a feature particularly relevant in fibrotic diseases of the liver and myocardium (32, 97, 139, 141, 151, 152). In this context, FAK inhibitors (e.g., defactinib, GSK2256098 and APG-2449) or YAP nuclear translocation inhibitors (e.g., Sitagliptin or Verteporfin) can reduce macrophage-driven inflammation and fibrosis. Furthermore, mechanical stress can activate the NLRP3 inflammasome in macrophages, promoting pyroptosis and IL-1β release which has been implicated in ventilator-induced lung injury, cardiac pressure overload, and hepatic fibrogenesis (54, 153, 154). Small-molecule inhibitors such as MCC950 or modulators targeting PDK1 can represent a promising therapeutical option to attenuate this mechanosensitive inflammatory axis (155, 156). Therapeutic strategies that restore oxidative metabolism, such as metformin or 2-deoxyglucose (2-DG), can counteract force-induced macrophage dysfunction by modulating macrophage metabolism and polarization (157). Finally, targeting the mechanical microenvironment itself through anti-fibrotic agents (e.g., Pirfenidone or Nintedanib) offers an indirect yet powerful strategy to modulate macrophage activity, as already demonstrated in a preclinical model of idiopathic pulmonary fibrosis (IPF) (158).

Altogether, this evidence underlines the importance of mechanical cues as active regulators of macrophage behavior that critically shape immune responses across organs. Understanding macrophages’ response offers a new dimension to our comprehension of tissue-specific immunity and pathogenesis. As the field evolves, targeting mechanotransduction pathways holds substantial promise for the development of precision immunotherapies aimed at blocking or reversing chronic conditions like inflammation and fibrosis.

## 4 Limitations and future directions

Macrophage mechanotransduction is a rapidly expanding field that warrants further investigation to explore its implications as therapeutic targets for various pathological conditions. Most studies have primarily focused on the mechanical regulation of macrophages toward a pro−inflammatory phenotype. However, macrophages exhibit high heterogeneity and multifunctionality; their phenotypes are far more complex than a simple M1/M2 dichotomy. Mechanical forces not only bias macrophages toward pro− or anti−inflammatory programs but also engage broader processes, such as metabolic reprogramming (glycolysis/oxidative phosphorylation shifts), lipid metabolism, efferocytosis, and proliferation that together determine functional outcomes. Mechanistically, substrate stiffness, stretch and topography are sensed via integrin-FAK-Src complexes and focal−adhesion maturation, which activate RhoA/ROCK−driven actomyosin contractility; mechanosensitive ion channels (e.g., Piezo1, TRPV4) mediate Ca2+ influx and downstream NFAT/CaMK signaling; and the LINC complex transmits cytoskeletal forces to the nucleus to modulate nuclear envelope tension. These inputs converge on mechanosensitive transcriptional regulators (YAP/TAZ, MRTF−A) and canonical inflammatory pathways (TLR4, MyD88, NF−κB), producing context−dependent transcriptional programs rather than binary states.

Understanding these alternative pathways regulated by mechanical activation will unveil potential therapeutic targets for manipulating macrophage function. The effect of mechanical forces on macrophage phenotype remains context−dependent: discordant results likely reflect differences in macrophage origin/subsets, substrate chemistry and adsorbed protein layers, force modality/intensity and duration, and concurrent soluble cues. Further research that systematically varies these parameters is necessary to elucidate the complex relationship between mechanical activation and macrophage phenotype.

The dynamic interplay between mechanical forces and cell to cell communication is an intriguing area of investigation. Macrophages engage in bidirectional signaling with parenchymal cells, fibroblasts, endothelial cells and other immune cells; mechanical modulation of integrin/TLR and ion−channel signaling can alter cytokine/chemokine outputs and efferocytosis, thereby reshaping tissue responses. Dissecting how mechanical cues influence these communication axes in defined *in vivo* contexts will clarify roles in homeostasis and disease.

Over the years, studies on the effects of mechanical forces have focused on acute cellular responses. Emerging evidence indicates that chronic mechanical perturbation can induce stable changes in transcriptional programs and chromatin architecture. For example, via Src−dependent histone modifications, altered accessibility of inflammatory loci, or persistent YAP/TAZ−mediated transcriptional states. Investigating the mechanisms, durability, and reversibility of mechanically induced epigenetic remodeling (histone marks, DNA methylation, chromatin accessibility) and their metabolic correlates represents a promising direction with direct translational implications for chronic inflammation and fibrosis.

## Data Availability

The original contributions presented in the study are included in the article/supplementary material. Further inquiries can be directed to the corresponding authors.
